# Biotechnological approaches to enhance salidroside, rosin and its derivatives production in selected *Rhodiola* spp. in vitro cultures

**DOI:** 10.1007/s11101-014-9368-y

**Published:** 2014-06-21

**Authors:** Marta Grech-Baran, Katarzyna Sykłowska-Baranek, Agnieszka Pietrosiuk

**Affiliations:** Department of Pharmaceutical Biology and Medicinal Plant Biotechnology, Faculty of Pharmacy, Medical University of Warsaw, Banacha 1 St., 02-097 Warsaw, Poland

**Keywords:** Biotransformation, In vitro cultures, *Rhodiola* spp., Rosin derivatives, Salidroside

## Abstract

*Rhodiola* (Crassulaceae) an arctic-alpine plant, is extensively used in traditional folk medicine in Asian and European countries. A number of investigations have demonstrated that *Rhodiola* preparations exhibit adaptogenic, neuroprotective, anti-tumour, cardioprotective, and anti-depressant effects. The main compounds responsible for these activities are believed to be salidroside, rosin and its derivatives which became the target of biotechnological investigations. This review summarizes the results of the diverse biotechnological approaches undertaken to enhance the production of salidroside, rosin and its derivatives in callus, cell suspension and organ in vitro cultures of selected *Rhodiola* species.

## Introduction

*Rhodiola* spp., herbaceous perennial plants of the Crassulaceae family, are extensively used in traditional medicine in Asian and European countries (Tolonen et al. 2003; Yousef et al. 2006; Galambosi et al. 2010; Panossian et al. 2010). The *Rhodiola* species grows in regions of cool temperature, in the sub-arctic areas of the northern hemisphere, including North and Central Europe, Asia and North America (GBIF 2010 http://www.gbif.org; Galambosi 2006; Guest and Allen 2014). The genus *Rhodiola* is believed to originate from the mountainous regions of Southwest China and the Himalayas and nowadays is distributed in mountainous as well as coastal habitats (Brown et al. 2002).

The current taxonomic status of the genus *Rhodiola* is quite complex due to the generally similar morphology (Brown et al. 2002; Liu et al. 2013). According to GBIF (2010) the genus *Rhodiola* comprises of 136 accepted species while the Plant List (http://www.theplantlist.org) includes 135 scientific plant names of species rank for the genus *Rhodiola*. Of these 61 are accepted species names, 16 have not been clarified.

The morphologicals of *Rhodiola* plants are as follows: stems dimorphic with usually very stout caudex or rhizome, usually with brown or blackish, membranous, scalelike leaves, sharply differentiated from much more slender, erect or ascending, leafy flowering stems (Liu et al. 2013). The plant is dioecious with male and female flowers located on different plants, and rarely hermaphroditic (Tutin 1964). Among the distinguishing characters of the genus are two series of stamens totaling twice the number of petals; free or nearly free petals (not joined in a tube); a stout rhizome from whose axils the flowering stems rise (Flora of China 2001).

Recently, genotyping and chemotaxonomic markers have been used to identify *Rhodiola* species within the genus. On the basis of the chemical profiles of the 47 collected *Rhodiola* samples of *R. crenulata, R. sachalinensis, R.* *himalensis, R. serrata, R. rosea, R. kirilowii* and *R. fastigiata* it was demonstrated that different kinds of characteristics reference markers occurred at various concentrations in the different *Rhodiola* species. The eight compounds: rosarin, rosavin, and rosin, tyrosol and salidroside, catechin, rhodionin and gallic acid have been proposed as reference chemotaxonomic markers. Salidroside and gallic acid were found in all species while rosarin and rosin were detected in *R. sachalinnsis, R. himalensis, R. rosea*. Rosavin was characteristic only for *R. himalensis, R. serrata, R.* *rosea* (Liu et al. 2013).

The roots and rhizomes of *Rhodiola* spp. have been reported to contain distinct groups of chemical compounds (Table 1). Initially, in the 1970s, the compound responsible for its unique pharmacological properties was believed to be salidroside (Saratikov and Krasnov 1987). According to the Russian Pharmacopeia (1989), the raw material of *R. rosea* should contain 0.8 % salidroside (Furmanowa et al. 1999). However, further studies revealed that not only salidroside but also rosin derivatives are important bioactive compounds (Sokolov et al. 1985, 1990; Wagner et al. 1994; Zapesochnaya et al. 1995). Wiedenfeld et al. (2007a) summarized the results of comparative studies on the activities of salidroside and rosin derivatives by Sokolov et al. (1985, 1990) which showed CNS, adaptogenic and immunostimulating properties, however he concluded that total *Rhodiola* *rosea* extracts are superior to the single components which indicates that the glycosides mentioned are not the only compounds responsible for the medicinal effect. In the subsequent studies it was reported that aqueous and hydroalcoholic extracts of *R. rosea* exhibited stronger neovascular reaction than rosavin applied alone (Skopińska-Różewska et al. 2008b).

**Table 1:** Chemical composition of *Rhodiola* spp.

Plant species	Compound	References
*R. crenulata* (in total over 26 compounds)	Salidroside and tyrosol, 2-(4-hydroxyphenyl)-ethyl-*O*-b-d-glucopyranosyl-6-*O*-β-d-glucopyranoside, *p*-hydroxyphenacyl-β-d-glucopyranoside, icariside D2, rutin, picein, lotaustralin, rodiocyanoside A, crenulatin, rhodionin, rhodiosin, daucosterol, β-sitosterol, hydroxycinnamic, gallic acid, creosides I, II, III, IV, and V	Wang and Wang (1992), Du and Xie (1995), Peng et al. (1995), Su et al. (2007), Nakamura et al. (2008), Chen et al. (2012)
*R. kirilowii* (in total over 49 compounds)	Salidroside and tyrosol, rosin, rosavin, rosarin, cinnamyl alcohol, herbacytrin, umbeliferon, esculetin, luteolin, tricetin, epigallocatechin, epigallocatechin gallate, lotaustralin, rodiocyanoside A, tannins, daucosterol and β-sitosterol, hydroxycinnamic, gallic acid, chlorogenic acid	Kang et al. (1992), Krasnov et al. (1978), Krajewska-Patan et al. (2006), Wiedenfeld et al. (2007b), Zuo et al. (2007), Wong et al. (2008), Krajewska-Patan et al. (2009), Krajewska-Patan et al. (2013)
*R. quadrifida*	Salidroside and tyrosol, rosin, rosavin, rosarin, cinnamyl alcohol, rhodiooctanoside, rhodiolin, mongrhoside, rhodiocyanosides A and B, rhodioflavonoside, rhodiooctanoside, tricetin, l-rhamnopyranoside, osmaronin, chlorogenic acid	Yoshikawa et al. (1996), Altantsetseg et al. (2007), Wiedenfeld et al. (2007a)
*R. rosea* (in total over 140 compounds)	salidroside and tyrosol, rosin, rosavin, rosarin, cinnamyl alcohol, epigallocatechin, epigallocatechin gallate, lotaustralin, rodiocyanoside A, herbacetin, kaemferol, rosiridol and rosaridin, daucosterol and β-sitosterol, Rhodiolosid A- C, organic acids, tannins, waxes, fats, proanthocyanidins, sachaliside, gallic, hydroxycinnamic acid, acetylrodalgin and tricin	Saratikov et al. (1967), Kurkin et al. (1985, 1986), Kurkin and Zapesochnaya (1986), Akgul et al. (2004), Yousef et al. (2006), Altantsetseg et al. (2007), Ali et al. (2008), Ma et al. (2013)
*R. sachalinensis* (in total over 44 compounds)	salidroside and tyrosol, rosarin, rosavin, cinnamyl alcohol, multiflorin B, tricetin, afzelin, kaempferol, rhodionin, rhodiosin, gallic acid, sachalosides I –V, sacranoside A, rhodiocyanoside A, lotaustralin, hetorodendrin, -glucopyranoside	Lee et al. (2000), Nakamura et al. (2007), Zhang et al. (2007a), Choe et al. (2012)

*Rhodiola rosea* extracts used in most pharmacological studies were standardized to a minimum 3 % of rosin and its derivatives and 0.8–1 % salidroside because the naturally occurring ratio of these compounds in *R*. *rosea* root is approximately 3:1 (Brown et al. 2002).

In numerous in vitro and in vivo studies on cells and animals, the extracts or pure salidroside have been examined and have shown strong biological activity. The main effects described to date are the following: adaptogenic and stress protective (Darbinyan et al. 2000; Spasov et al. 2000; De Bock et al. 2004; Olsson et al. 2009), antioxidant (Chen et al. 2009b; Schriner et al. 2009; Calcabrini et al. 2010; Mao et al. 2010), anti-tumour (Skopińska-Różewska et al. 2008a, 2008c, Hu et al. 2010a, b; Sun et al. 2012), anti-depressive (Chen et al. 2009a, Diermen et al. 2009), neuroprotective (Zhang et al. 2007b, Yu et al. 2008, Chen et al. 2009a, Yu et al. 2010), cardioprotective (Wu et al. 2009, Cheng et al. 2012) hepatoprotective (Nan et al. 2003), and immunostimulating (Seo et al. 2001, Wójcik et al. 2009, Siwicki et al. 2012). Also the cinnamyl alcohol derivate rosavin shows a stimulating effect on the CNS (Wagner et al. 1994), demonstrated as spontaneous motor action and antihypnotic properties (Sokolov et al. 1985, 1990). Other investigations revealed immunostimulating and anticancer effects of rosavin (Zapesochnaya et al. 1995; Skopińska-Różewska et al. 2008b).

Therefore *Rhodiola* preparations may be therapeutically applied to humans, for preventing or treating disorders like neurodegenerative diseases, cerebral ischaemia, fatigue, hypoxia, diabetes, and cancer, among many others.

Phytochemical investigations of *Rhodiola* plants pointed out that the content of salidroside, rosin and its derivatives depends on the morphological part of the plant—higher in rhizomes than in roots (Przybył et al. 2008; Węglarz et al. 2008; Tasheva and Kosturkova 2010). It also depends on the age and sex of the plant—rhizomes originated from male plants accumulated higher amounts of salidroside (Revina et al. 1976; Platikanov and Evstatieva 2008; Węglarz et al. 2008; Kucharski et al. 2011). Salidroside content in plants is also connected with the place and time of harvesting (Revina et al. 1976; Kurkin et al. 1985; Bykov et al. 1999; Buchwald et al. 2006) as well as place of plant origin (Altantsetseg et al. 2007; Węglarz et al. 2008). To date, the results from the papers concerning chemical profiling of *Rhodiola* plants demonstrated the higher salidroside and rosin and its derivatives content in plants collected from natural sources than derived from field cultivation (Table 2).

**Table 2:** Content of salidroside, rosin and its derivatives (% DW) in *Rhodiola* species

Genus	Natural source		Plantation		References
	Salidroside content (%)	Rosin and its derivatives content (%)	Salidroside content (%)	Rosin and its derivatives content (%)	
*Rhodiola crenulata*	0.1–2.05	Not detected	No data	No data	Abidov et al. (2003), Chen et al. (2012),
*Rhodiola kirilowii*	0.32–2.5	Not detected	0.0002–0.005	0.03 (total content of rosin, rosavin, rosarin)	Krajewska-Patan et al. (2009), Tao et al. (2010), Zhou et al. (2011), Gryszczynska et al. (2012)
*Rhodiola quadrifida*	0.016–0.45	Rosin-0.26–0.31 Rosavin-0.19–0.76 Rosarin-0.26–1.1	No data	No data	Yoshikawa et al. (1996), Altantsetseg et al. (2007), Wiedenfeld et al. (2007a)
*Rhodiola rosea*	0.14–1.76	Rosin-0.1–0.31 Rosavin-0.4–3.7 Rosarin-0.08–1.1	0.14–1.6	Rosin-0.36–0.53 Rosavin-0. 9–5 rosarin-0.16–0.3	Kurkin et al. (1985, 1986), Galambosi (2006), Altantsetseg et al. (2007), Platikanov and Evstatieva (2008), Bozhilova (2011), Kołodziej and Sugier (2012), Peschel et al. (2013), Saunders et al. (2013)
*Rhodiola sachalinensis*	0.1–1.5	Rosin-No data Rosavin-0.034 Rosarin-0.0097	0.1–0.5	No data	Li et al. (2001), Yan et al. (2004), Nakamura et al. (2007), Choe et al. (2012)

*DW* dry weight

Research studies on *Rhodiola* species have been stimulated by their documented pharmacological properties. Recently a sharp increase in the demand for *Rhodiola*-based products has been observed worldwide which is connected with the necessity of a stable raw material supply and—at the same time—extensive collecting from natural sources. *Rhodiola rosea* is the most exploited species for commercial purposes. Due to the intensive collection, natural populations of *R. rosea* are highly threatened and included in the list of endangered plant species in many countries (Russia, Great Britain, the Czech Republic, Bosnia and Herzegovina, vulnerable in Slovakia; in Bulgaria collection is strictly forbidden; in Poland *R. rosea* is only found in National Parks) (Galambosi 2006; Kołodziej and Sugier 2012). Sufficient yields of field-cultivated *R. rosea* roots/rhizomes could be obtained within 5–7 years of cultivation, however the costs are high (Galambosi 2006; Peschel et al. 2013). Another approach to provide a stable supply of the desired compound is its chemical synthesis. The chemical synthesis of rosavin, the accepted marker for genetically pure *R. rosea* and its extracts (Brown et al. 2002), was performed by Patov et al. 2006. In the case of salidroside the procedure of it synthesis in a multikilogram scale with 72 % overall yield and >98 % purity has been developed recently by Shi et al. (2011). Nevertheless chemical synthesis seems not to be the solution in the case of *Rhodiola* preparations as it is obvious on the basis of pharmacological studies that salidroside and rosavins are not the only biologically active ingredients responsible for its efficacy and health benefits (Brown et al. 2002; Nan et al. 2003; Zuo et al. 2007; Panossian et al. 2010; Gupta et al. 2010; Choe et al. 2012; Wang et al. 2012; Chen et al. 2012).

Biotechnological methods offer the possibilities to produce useful compounds under controlled conditions independent of environmental changes and elaboration of scale-up procedures would reduce labour costs and improve productivity of secondary metabolites. Moreover, the cells and organs involved directly with the biosynthesis of compounds of interests could be cultivated and manipulated to enhance their production.

Numerous studies have revealed that the addition of an elicitor (microbial, physical, or chemical factors) or/and a precursor to the culture media significantly increases the production of secondary metabolites (Kolewe et al. 2008; Shilpa et al. 2010; Vijaya et al. 2010).

This review discusses the ways to enhance salidroside and rosin and its derivatives production in various types of in vitro cultures of *Rhodiola* spp. To date, to the in vitro cultures have been introduced mainly for *R. rosea* and selected Asian species, such as *R. crenulata,* *R. kirilowii*, *R. quadrifida* and *R. sachalinensis*. Table 3 presents the distribution of the species.

**Table 3:** Natural occurrence of *Rhodiola* species

Genus	Examples of occurrence	References
*Rhodiola crenulata* (Hook. f. et Thoms) H. Ohba	Asia (Tibet, China)	Wang and Wang (1992), Peng et al. (1995), Chen et al. (2012)
*Rhodiola kirilowii* (Regel) Regel et Maximowicz	Asia (Tibet-Qinghai), Europe (Poland)	Komarov and Juzepczyk (1939), Krajewska-Patan et al. (2008b)
*Rhodiola quadrifida* (Pall) Fish. and May.	Asia (China, Tibet, Mongolia)	Yoshikawa et al. (1996), Altantsetseg et al. (2007), Wiedenfeld et al. (2007a)
*Rhodiola rosea* L.	Asia (China), North America (USA, Canada) Europe (Russia, Poland, Scandinavia, Sweden)	Galambosi (2006), Panossian et al. (2010), Hung et al. (2011), Kołodziej and Sugier (2012), Saunders et al. (2013)
*Rhodiola sachalinensis* A Bor	Eastern Europe, Asia (China, Korea)	Lee et al. (2000), Li and Chen (2001), Yan et al. (2004)

## Biosynthetic pathway of salidroside, rosin and its derivatives

**Fig. 1:**
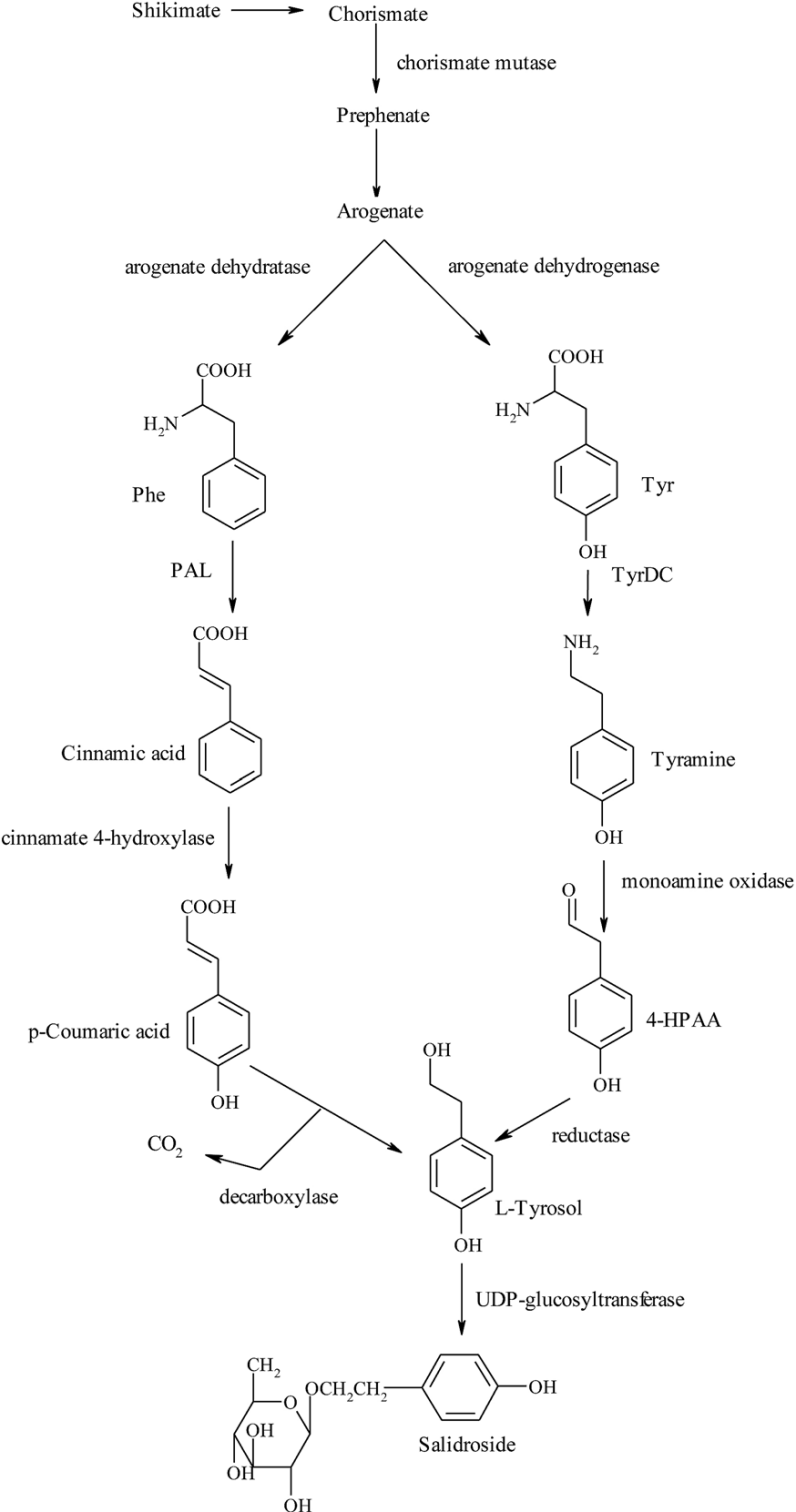
Biosynthetic pathway of salidroside (Ma et al. 2008). *Phe* l-phenylalanine, *Tyr* l-tyrosine, *PAL* phenylalanine ammonia-lyase, *TyrDC* tyrosine decarboxylase, *4-HPAA* 4-hydroxyphenylacetaldehyde

The biosynthetic pathway of the salidroside precursor l-tyrosol and its regulation are not completely understood. There are two different views in the literature concerning the production of salidroside. Both are connected with the shikimate pathway producing l-phenylalanine or l-tyrosine (Yao et al. 1995). The first is that tyrosol is presumably produced by a decarboxylase from p-coumaric acid, which is mainly derived from l-phenylalanine (Xu and Su 1997). The second view states that the precursor of tyrosol may be tyramine, which is synthesized from tyrosine (Zhang et al. 2011) (Fig. 1).

In order to elucidate the molecular pathways involved in tyrosol biosynthesis Ma et al. (2008) analyzed the effect of overexpressing the endogenous PALrs1 gene in *R. sachalinensis* on the production of higher levels of p-coumaric acid and salidroside. The obtained results showed that overexpression of the PALrs1 gene resulted in a 3.3-fold increase of p-coumaric acid. However, the levels of tyrosine, tyrosol and salidroside were 2.6-fold, 4.7-fold and 7.7-fold lower, respectively, in PALrs1 transgenic plants than in controls. Overexpression of the PALrs1 gene and accumulation of p-coumaric acid did not facilitate tyrosol biosynthesis. Thus, tyrosol probably is not derived from l-phenylalanine. Following the second view of salidroside biosynthesis Gyorgy et al. (2009) analyzed the expression of the *R. rosea* TyrDC gene encoding tyrosine decarboxylase. TyrDC expression was detected in leaves as well as in roots. The expression was significantly higher in roots, which coincides with the fact that salidroside accumulates preferentially in the underground parts of the plant. The expression was also higher in the line accumulating high levels of salidroside, compared to the line with lower salidroside content. Similar results were obtained by Zhang et al. (2011). In this study, a cDNA encoding TyrDC was isolated from *R. sachalinensis* and the effects of recombinant RsTyrDC and sense and antisense overexpression of endogenous RsTyrDC on tyrosol synthesis were investigated. For sense-RsTyrDC expression, RNA gel blotting indicated that RsTyrDC was over-expressed at transcriptional levels and TyrDC activity in the RsTyrDC transgenic lines was 74 and 127 % higher, respectively, than in the empty vector-transformed control plants. This overexpression resulted in the accumulation of tyrosol and salidroside. Simultaneously there was evidence of reduced accumulation of the endogenous TyrDC transcripts of *R.* *sachalinensis* in the antisense plant.

Summarizing these research results, it is tempting to speculate that the biosynthesis of salidroside begins with the decarboxylation of tyrosine by TyrDC, which produces tyramine.

**Fig. 2:**
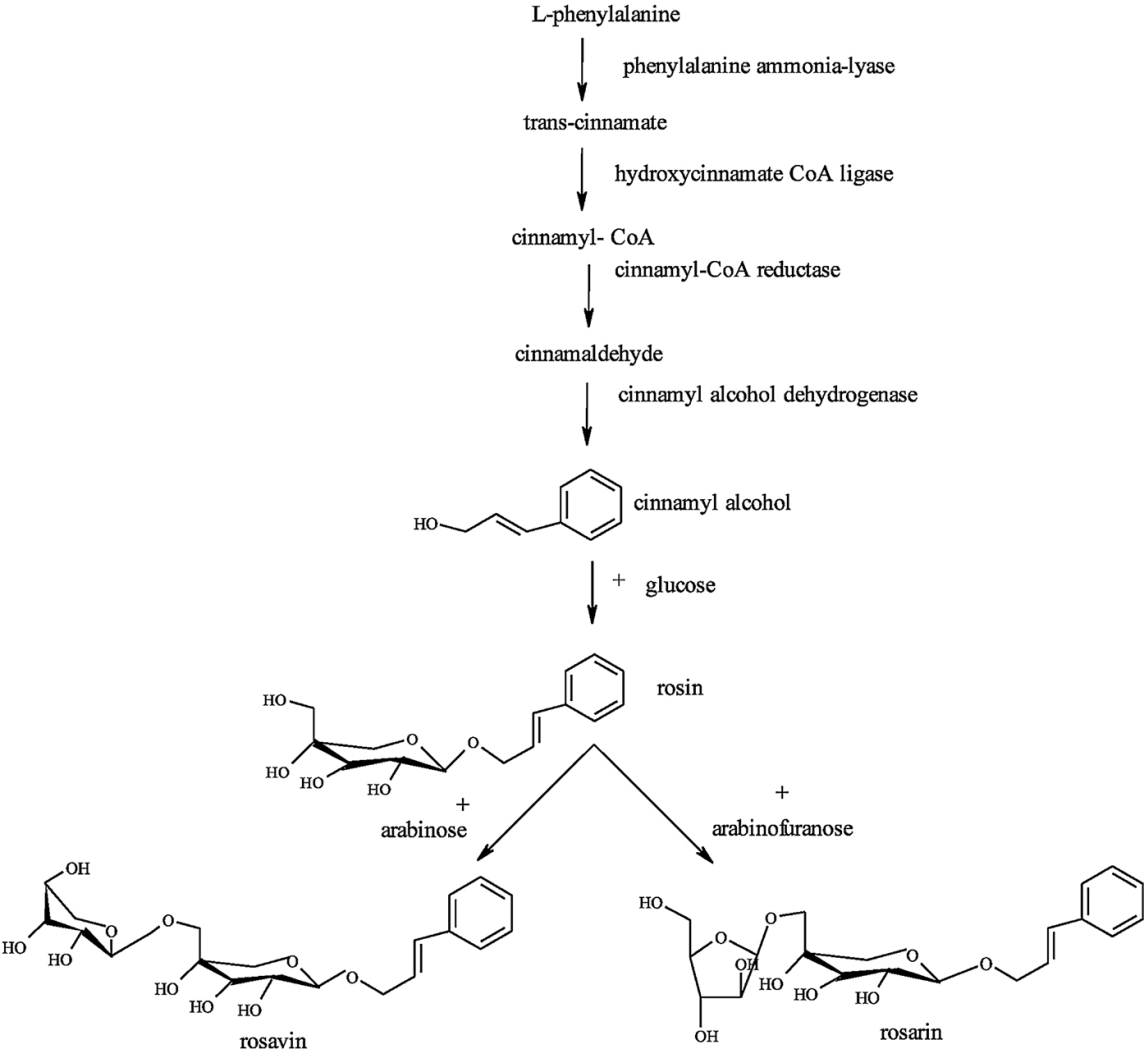
Biosynthetic pathway of rosin, rosavin and rosarin (György 2006)

The production of rosin and its derivatives in *Rhodiola* is restricted to only a few species. Biosynthesis of phenolic glycosides occurs spontaneously in *Rhodiola* roots and rhizomes (Krajewska-Patan et al. 2013). According to György (2006) the cinnamyl alcohol glycosides are products of phenylpropanoid metabolism, derived from l-phenylalanine. Phenylalanine ammonia lyase (PAL) converts l-phenylalanine to cinnamic acid. From cinnamic acid cinnamyl-CoA ester is formed through hydroxycinnamate: CoA ligase (4CL). This CoA ester is reduced to cinnamaldehyde by cinnamyl-CoA reductase (CCR). The cinnamaldehyde is further reduced by cinnamyl alcohol dehydrogenase (CAD) to cinnamyl alcohol (Fig. 2). The enzymes that take part in the formation of the glycosides of cinnamyl alcohol are not yet described. By one glucose transfer rosin is formed, which is the simplest cinnamyl alcohol glycoside. From rosin rosavin is formed by adding an arabinopyranose unit and rosarin by adding an arabinofuranose unit. Further glycosides may be formed depending on the sugar type and the connection site.

## The enhancement of salidroside, rosin and its derivatives production in *Rhodiola* spp. in vitro cultures

Biotechnology offers an opportunity to exploit cells, tissues, organs or entire plants by growing them in vitro and to genetically manipulate them to get the desired compounds (Rao and Ravishankar 2002). Till now the biotechnological production of salidroside using plant cell cultures derived from *R. crenulata, R. rosea, R. kirilowii, R. quadrifida* and *R.* *sachalinensis* have been investigated. The influence of external factors such as nutrient levels, stress factors, light and growth regulators on salidroside and rosavins accumulation is summarized in Table 4.

**Table 4:** Salidroside, rosin and its derivatives production in plant cell, tissue and organ cultures of selected *Rhodiola* spp.

Plant species	Culture type	Medium	Growth regulators	Product	References
*R. crenulata*	Callus culture and cell suspension culture	MS	3.0 mg/l NAA, 0.5 mg/l BA	Salidroside (26.48 mg/g DW)	Shi et al. (2013)
		MS	0.5 mg/l TDZ, 0.5 mg/l NAA		
*R. kirilowii*	Callus culture	MS	2.0 g/l BA, 1.0 g/l adenine chloride, 2.0 g/l NAA or 2,4-D	Salidroside (0.16 mg/g DW)	Krajewska-Patan et al. (2009)
*R. quadrifida*	Callus culture	MS	1 mg/l 2,4-D, 0.1 mg/l 6-BA, 0.5 mg/l KT	Salidroside (2.8 mg/g DW)	Sheng et al. (2005)
*R. rosea*	Shoots and rooted plantlets culture	NN	0.01 mg/l NAA and 0.1 mg/l IAA	Salidroside Rosin and its derivatives (content not showed)	Furmanowa et al. (1995)
	Callus culture	MS	2 mg/l BAP, 2 mg/l NAA, 1 g/l, casamino acids	Salidroside Rosin and its derivatives (content not showed)	Furmanowa et al. (1998)
	Shoot culture	MS	2 mg/l zeatin or 2 mg/BA	Salidroside (5.3–6.4 mg/g DW)	Tasheva and Kosturkova (2010)
	Rooting induction	½ MS	2 mg/l IBA, 0.2 mg/l IAA		
*R. sachalinensis*	Compact callus aggregate culture	MS	0.3 mg/l NAA, 3 mg/l BA	Salidroside (6.8 mg/g DW)	Xu et al. (1998c)
*R. sachalinensis*	Compact callus aggregate culture Callus culture Compact callus aggregate culture	MS MS MS	0.2 mg/l NAA, 3.3 mg/l BA, 40 g/l of sucrose 5 mg/l BA, 2 m/l IBA 5 mg/l BA, 2.5 mg/l IBA, 2.0 mg/l 2,4-D	Salidroside (7.9 mg/g DW) Salidroside (5.09 mg/g DW) Salidroside (41.94 mg/g DW)	Xu et al. (1999) Wu et al. (2003)

*DW* dry weight

## The effect of growth regulators

Enhanced salidroside accumulation in *R. sachalinensis* compact callus aggregate culture (CCA) was observed by Xu et al. (1998c) compared to dispersed cells. The CCA were maintained on MS medium supplemented with 3 % sucrose, 0.3 mg/l NAA, and 3 mg/l BA. The salidroside yield in CCA after 24 days of culture was 6.8 mg/g DW and, approximately six times as much as that of the dispersed cells; the growth rate of CCA was comparable to that of the dispersed cells. Furthermore, the suitability of airlift reactors for large-scale culture of CCA was investigated in a 5-l airlift reactor. The salidroside amount was similar to that of shake-flask cultures though the biomass was relatively low. Xu et al. (1999) estabished liquid cultures of compact callus aggregates (CCA) of *R.* *sachalinensis* derived from stems, and found that a higher cytokinin/auxin ratio was beneficial for both CCA growth and salidroside accumulation. The maximum salidroside yield of 4.7 mg/g DW was achieved when MS medium was supplemented with 0.2 mg/l NAA and 3.3 mg/l BA. Also a favourable effect on the metabolite productivity of raising the initial sucrose levels has been observed. The authors indicated that for growth and salidroside accumulation (up to 7.9 mg/g DW) the CCA culture should be cultivated in MS medium containing 40 g/l of sucrose, 0.2 mg/l NAA and 3.3 mg/l BA. A compact callus aggregate culture (initiated from callus established from cotyledons) of *R. sachalinensis* reached a high yield of salidroside under appropriate culture conditions. A significant increase in salidroside content (up to 10.78 mg/g DW) was obtained when the inoculum amount was 10 % and the concentration of BA and IBA added to the liquid medium was 5 and 2.5 mg/l, respectively. The addition of 2,4-D at a concentration of 2.0 mg/l boosted the salidroside accumulation in the treated cells to 41.94 mg/g DW while the addition of kinetin inhibited both salidroside accumulation and growth of the CCA suspension culture (Wu et al. 2003).

Callus tissue of *R. crenulata* was initiated from three types of explants: leaf, stem and root, that were maintained on half strength MS medium supplemented with 3.0 mg/l NAA and 0.5 mg/l BA. Callus initiation was noticeably faster on leafy explants (within 9 days from the beginning of culture) compared to stems and roots (13 and 15 days, respectively). For callus subcultures and subsequent cell suspension cultures full strength MS containing 0.5 mg/l TDZ (thidiazuron) and 0.5 mg/l NAA proved to be the best. The average salidroside content in cell suspension culture reached 26.48 mg/g DW (Shi et al. 2013).

A callus culture of *R. kirilowii* obtained by Krajewska-Patan et al. (2009) also produced salidroside. Two lines of callus tissue were initiated. The first started from cotyledons on MS medium supplemented with BAP 2.0 mg/l, adenine chloride 1.0 mg/l and NAA 2.0 mg/l. The second started from hypocotyles cultivated on MS supplemented with 1.0 mg/l 2,4-D. The results of phytochemical analyses revealed that callus tissues cultivated on solid medium produced the complete spectrum of active compounds characteristic for the roots of the plant. The highest salidroside content of 0.166 mg/g DW was observed in the line obtained from cotyledones.

An investigation concerning the effect of plant growth regulators on the induction and proliferation of callus of *Rhodiola quadrifida* was conducted on MS medium. The medium containing 1 mg/l 2,4-D, 2 mg/l NAA, 0.5 mg/l BAP and 0.1 mg/l kinetin proved to be the best for induction of the callus from *R.* *quadrifida* (the induction rate was 83.3 %). The optimized combination of plant growth regulators for callus subculture was 1 mg/l 2,4-D, 0.1 mg/l BAP and 0.5 mg/l kinetin. When the callus was cultured in the optimum medium for 30 days the salidroside content reached 2.8 mg/g DW (Sheng et al. 2005).

Furmanowa et al. (1995) reported the micropropagation of *R. rosea* by apical and axillary meristems. Out of many combinations of growth regulators added to different basal media the best for shoot development were 0.1 mg/l kinetin and 0.1 mg/l IAA added to NN medium (Nitsch and Nitsch 1969). The shoots were rooted on the same medium. In the shoots growing in vitro traces of salidroside, rosin and its derivatives were found. In further studies Furmanowa et al. (1998) described the condition for induction and maintenance of *R. rosea* callus cultures. Green callus tissue was formed from leaf segments on MS medium containing 2 mg/l BAP, 2 mg/l NAA, 1 g/l casamino acids and 6 % sucrose. Following four passages, callus was subcultured on new media with various combinations of auxins and cytokinins to find the best growth promoting conditions. Two lines were obtained: deep green and yellow. The phytochemical analyses revealed that only one green line produced salidroside, however, traces of rosin and its derivatives were present in all three types of investigated tissues.

In vitro cultures for micropropagation and the reintroduction of *R rosea* were established by Tasheva and Kosturkova (2010). The authors reported that the best shoot induction was obtained from seedling explants on media containing 2 mg/l zeatin or BA each. Efficient rooting was induced on half-strength MS containing 2 mg/l IBA and stimulated by adding 0.2 mg/l IAA. Regenerants rooted in perlite, peat, and soil (1:1:2), adapted in a greenhouse, and transplanted in the mountains survived (70 %) and developed similarly to the wild plants. The salidroside content of these plants after one or 2 years reached 6.4 and 6.1 mg/g DW in rhizomes and 6.2 and 5.3 mg/g DW in roots.

## Precursor feeding and elicitation

Plant cells are structures accumulating many chemical compounds that take part in biochemical pathways of growth, defense, and survival. However, plant secondary metabolites often occur in plants in very small amounts, less than 1 % of dry weight (Oksman-Caldentey and Inze 2004). Hence, biotechnology offers an opportunity to exploit the cells, tissues, organs or entire organisms by growing them in vitro and to genetically manipulate them to obtain the desired compounds (Ramachandra Rao and Ravishankar 2002).

Elicitation and precursor feeding in *Rhodiola* spp. were reported by Xu et al. (1998a), using *R.* *sachalinensis* cell suspension cultures. Among the examined elicitors, one extracted from *Aspergillus niger* was the most effective for salidroside production which reached up to 9.9 mg/g DW. In comparison untreated cultures produced only 1.7 mg/g DW of salidroside. The applied precursor feeding strategy revealed that a significantly high level of salidroside 14.4 mg/g DW was obtained when tyrosol was added to the medium. The combined application of both precursor and elicitor feeding resulted in a markedly high salidroside content of 17.3 mg/g DW (Xu et al. 1998a). Table 5 summarizes studies on elicitors and precursors applications for the production of phenolic glycosides in different *Rhodiola* spp.

**Table 5:** Elicitors and precursors used for the production of phenolic glycosides in *Rhodiola* species

Plant species	Culture type	Elicitor	Precursor	Product	References
*R. kirilowii*	Callus culture		l-Tyrosol (2.5–5 mM)	Salidroside (11 mg/g DW at 2.5 mM)	Krajewska-Patan et al. (2008a)
	Natural root culture Hairy root culture		l-Tyrosol (2.5 mM) l-Tyrosol (2.5 mM)	Salidroside (21.9 mg/g DW on the day of the highest TGase activity) Salidroside (7.5 mg/g DW on the day of the highest TGase activity)	Grech-Baran et al. (2013)
	Natural root culture Natural root culture Natural root culture Hairy root culture Hairy root culture Hairy root culture		Trans-cinnamyl alcohol (2.5 mM) and/or 1 % sucrose Trans-cinnamyl alcohol (2.5 mM) and/or 1 % sucrose	Rosavin (6.0 mg/g DW) Rosarin (0.12 mg/g DW) Rosin (5 mg/g DW) Rosavin (9.8 mg/g DW) Rosarin (0.4 mg/g DW) Rosin (not detected)	Grech-Baran et al. (2014)
*R. rosea*	Cell suspension culture		Trans-cinnamyl alcohol (2.5 mM)	Rosavin (10.01 mg/g DW)	Furmanowa et al. (1999)
*R. rosea*	Compact callus aggregate culture		Trans-cinnamyl alcohol (0.05–5 mM)	Rosin (12.5 mg/g DW at 2 mM)	György et al. (2004)
	Compact callus aggregate culture		Trans-cinnamyl alcohol (0.05–5 mM)	Rosavin (0.5 mg/g DW at 0.1 mM)	
	Compact callus aggregate culture		Trans-cinnamyl alcohol (2 mM and 1 % glucose)	Rosin (5.7 mg/g DW) Rosavin (0.15 mg/g DW)	György et al. (2005)
	Callus aggregate culture (solid medium)		l-tyrosol (5 mM)	Salidroside (23.15 mg/g DW)	Krajewska-Patan et al. (2007a)
	Compact callus aggregate culture		l-tyrosol (2.0 or 5 mM)	Salidroside (43.22 mg/g DW at 5 mM)	
	Callus aggregate culture (hypocotyl strain)		Trans-cinnamyl alcohol (2.5 or 5 mM)	Rosin (10.6 mg/g DW at 2.5 mM) Rosarin (0.05 mg/g DW at 2.5 mM) Rosavin (0.63 mg/g DW at 2.5 mM) Salidroside (1.1 mg/g DW at 2.5 mM)	Krajewska-Patan et al. (2007b)
	Callus aggregate culture (axially buds strain)		Trans-cinnamyl alcohol (2.5 or 5 mM)	Rosin (8.5 mg/g DW at 2.5 mM) Rosarin (0.04 mg/g DW at 2.5 mM) Rosavin (0.93 mg/g DW at 2.5 mM Salidroside (0.58 mg/g DW at 2.5 mM)	
*R. sachalinensis*	Cell suspension culture	*Ganoderma lucidum* *Aspergillus niger* *Fusarium oxysporum* *Schizophyllum commune* *Polystictus versicolor* *Rhizopus delemar* *Penicillium* sp. (50 mg/l)		Salidroside (8.2 mg/g DW) Salidroside (9.9 mg/g DW) Salidroside (2 mg/g DW) Salidroside (2.2 mg/g DW) Salidroside (1 mg/g DW) Salidroside (2.6 mg/g DW) Salidroside (7.8 mg/g DW)	Xu et al. (1998a)
			l-Phenylalanine (0.05–1 mM)	Salidroside (1.9 mg/g DW at 0.05 mM)	
			l-Tyrosol (0.05–1 mM)	Salidroside (14.4 mg/g DW at 1 mM)	
			l-Tyrosine (0.05–1 mM)	Salidroside (10.1 mg/g DW at 1 mM)	
	Cell suspension culture		l-Tyrosol (1–3 mM)	Salidroside (154.9 mg/g DW at 3 mM)	Xu et al. (1998b)
	Compact callus	Salicylic acid		Salidroside (8.9 mg/g DW at 15 mg/l)	Wu et al. (2003)
	aggregate culture	(5–45 mg/l)	l-Phenylalanine	Salidroside (9 mg/g DW at 1 mM)	
			(0.5–3 mM)		
*R. sachalinensis*	Compact callus aggregate culture		l-Tyrosol (0.5–4 mM)	Salidroside (57.7 mg/g DW at 4 mM)	Wu et al. (2003)
			l-Tyrosine (0.5–4 mM	Salidroside (8.9 mg/g DW at 0.5 mM)	
	Hairy root culture	*Aspergillus niger* *Coriolus versicolor* *Ganoderma lucidum*		Salidroside (6.2 mg/g DW at 0.05 g/l) Salidroside (6 mg/g DW at 0.05 mg/l) Salidroside (6.5 mg/g DW at 0.05 g/l)	Zhou et al. (2007)
		(0.025–0.1 mg/l)			
			l-Phenylalanine (0.5–2 mM)	Salidroside (6.4 mg/g DW at 1 mM)	
			l-Tyrosol (0.5–2 mM)	Salidroside (6.6 mg/g DW at 1 mM)	
			l-Tyrosine (0.5–2 mM	Salidroside (6.8 mg/g DW at 1 mM)	

*DW* dry weight, *TGase* tyrosol-glucosyltransferase

In further studies, Xu et al. (1998b) showed that the biotransformation reaction depends not only on precursor addition, but also on the enzyme activities present in the cell suspension culture of *R. sachalinensis*. The relevant enzymes are classified as glucosyltransferases. The authors presented the effect of the tyrosol-glucosyltransferase (TGase) activity on tyrosol conversion to salidroside. The studies revealed that the low yield of salidroside in the cell culture of *R. sachalinensis* was connected with the low efficiency of glucosylation and non-synchronization between the TGase activity and tyrosol accumulation in cells. The highest TGase activity was found to be in the exponential growth phase on day 8. By repeated addition 3 mM tyrosol at 24 h intervals, starting on the day of the highest TGase activity, a significant salidroside yield of 154.5 mg/g DW was obtained (Xu et al. 1998b). To date, that investigation has produced the highest intracellular content of salidroside obtained through biotransformation by *R. sachalinensis* in vitro cultures.

The low efficiency of glucosylation and the lack of synchronization between TGase activity with tyrosol appearance are believed to be the main cause for low salidroside yield in cell suspension cultures of *R. sachalinensis* (Xu et al. 1998b). These findings prompted Ma et al. (2007) to research on glucosyltransferases on the molecular level. The putative UDP-glucose:tyrosol glucosyltransferase (UGT) cDNA *UGT73B6* was isolated and it was also indicated that it exists in a single copy in the *R.* *sachalinensis* genome. Moreover *UGT73B6* transcripts were more abundant in roots and callus than in stems and barely detected in leaves. The detected transcript levels were well correlated with the salidroside content where higher amounts of salidroside were found in roots and callus than in stems and leaves. Next *UGT73B6* was successfully transferred into *R. sachalinensis* by *Agrobacterium tumefaciens*. Overexpression of the *UGT73B6* gene resulted in an evident increase of salidroside content up to 2- and 2.4-fold in transgenic plants and transgenic calli, respectively, compared with the untransformed controls. These results suggested the role of *UGT73B6* in the conversion of tyrosol to salidroside in *R. sachalinensis*. However, *UGT73B6* expression was scarcely detectable in leaves with unchanged salidroside content which indicated the possibility that other UDP-glucosyltransferases might exist.

Further examinations lead to the isolation of two glucosyltransferase genes/cDNAs from roots and cells of *R.* *sachalinensis* cultured in the presence of methyl jasmonate: *UGT72B14* and *UGT74R1* (Yu et al. 2011). The transcripts of the former glucosyltransferase was more abundant in roots, while the latter glucosyltransferase was highly expressed in callus tissue. The relative activity of recombinant *UGT72B14, UGT74R1* and *UGT73B6* was investigated revealing the highest relative rates towards tyrosol, salicylic acid and p-coumaric acid, respectively. The overexpression of *UGT72B14, UGT74R1* and *UGT73B6* in hairy roots established with *A. rhizogenes* resulted in salidroside contents of about 19.81, 5.72 and 8.76 mg/g DW, respectively. Moreover, as expected the UGT73B6 hairy roots were capable of producing higher amounts of salidroside in comparison to *UGT73B6* transgenic plants or callus tissue reported by Ma et al. (2007).

The activity of tyrosol-glucosyltransferase (TGase) and the efficiency of salidroside production through biotransformation in natural and transformed root cultures of *R. kirilowii* were investigated by Grech-Baran et al. (2013). To induce the TGase activity, tyrosol 2.5 mM was added to natural and transformed root cultures on the day of inoculation. The first peak in TGase activity of 0.23 U/μg was detected on day 9 in the natural root culture, which correlated with the highest salidroside content of 15.8 mg/g DW. However, the highest TGase activity of 0.26 U/μg was noted on day 15. In the transformed root culture, on day 18 the highest TGase activity of 0.15 U/μg, was observed and coincided with the highest salidroside content of 2.4 mg/g DW. These findings prompted us to carry out supplementation of cultures with tyrosol on the days of the previously detected highest activity of TGase. The applied strategy resulted in a notable increase of salidroside production of up to 21.9 mg/g DW and 7.5 mg/g for the natural and transformed root cultures, respectively.

Wu et al. (2003) described the feeding of salicylic acid (SA) (a signaling compound acting in response to stress factors), l-phenylalanine (Phe), l-tyrosine (Tyr), l-tryptophan (Trp), and l-tyrosol in suspensions of compact callus aggregate (CCA) cultures of *R. sachalinensis*. Each precursor or SA was added during the callus inoculation time. The results implied that feeding of Trp did not increase the salidroside yield; nevertheless, the addition of SA, Phe, and Tyr at a concentration of below 15 mg/L increased the salidroside content slightly. Supplementation with tyrosol at a concentration of 4 mM resulted in a boosted production of salidroside up to 57.7 mg/g DW.

The approach of tyrosol addition into CCA cultures of *R. rosea* was also described by Krajewska-Patan et al. (2007a). The most pronounced results seen after the biotransformation were with the use of 5 mM tyrosol. The salidroside content obtained was comparable to the results described above for the *R. sachalinensi*s CCA culture.

Upon the challenge for increasing the secondary metabolite accumulation, *Agrobacterium rhizogenes* has been used for the induction of transgenic root cultures. Transformed root cultures are gaining increasing attention because of their fast growth, and genetic and biochemical stabilities (Giri et al. 2001). Thus, the biotransformation possibility in transformed root cultures of *R. sachalinensis* was studied by Zhou et al. (2007). The use of elicitors (*Aspergillus niger*, *Coriolus versicolor*, *Ganoderma lucidum*) and precursors (l-tyrosol, l-tyrosine, and l-phenylalanine) resulted in a significant increase of salidroside content, from 4.2 mg/g DW in the control to as much as 6.5 mg/g DW and 6.8 mg/g DW after treatment with the *Ganoderma lucidum* or l-tyrosine, respectively. The optimal concentration for elicitor and precursor in the liquid medium was 0.05 mg/l and 1.0 mM, respectively.

With the aim of enhancing the level of rosin and its derivatives through biotransformation in *R. rosea* cell suspension cultures, Furmanowa et al. (1999) measured the glycosylation level of exogenously applied trans-cinnamyl alcohol (CA) 2.5 mM. More than 90 % of the trans-cinnamyl alcohol, used as the substrate for biotransformation, was converted by cells of *R. rosea* to a number of constituents, including rosavin of up to 10.01 mg/g DW (1 %) of cell DW within 72 h. In studies involving *R. rosea* compact callus aggregate liquid culture, György et al. (2004) observed that optimal concentration of CA was 2 mM for rosin production with a yield of 12.5 mg/g DW. The highest rosavin amount of 0.83 mg/g DW was determined at the presence of 0.1 mM CA. Moreover, no rosarin was detected. Furthermore, the authors investigated the possibility of increasing the yield of biotransformation products by adding glucose to the culture medium (György et al. 2005). When the CCA liquid cultures were supplemented with 10 g glucose and 2 mM CA, the rosin content increased to 5.7 mg/g DW, and 4 new unexpected compounds related to rosin derivatives were detected. Nevertheless, the rosavin yield was still very low and reached a top concentration of only 0.02 % DW.

In order to investigate the biotransformation capacity of *R. rosea* CCA on solid medium CA was added at a concentration of 2.5 mM or 5 mM (Krajewska-Patan et al. 2007b). It is worth pointing out that the CCA maintained on solid medium produced the whole spectrum of active compounds present in the intact plant: salidroside, tyrosol, rosin and its derivatives, chlorogenic and gallic acids. The highest obtained rosin amount was 10.6 mg/g DW. Similar to prior studies, rosavin and rosarin were produced in low amounts.

During our investigation, we compared the rosin and its derivatives production by biotransformation in *R. kirilowii* natural and hairy root cultures (Grech-Baran et al. 2014). The obtained results confirmed that the addition of 2.5 mM exogenous CA enabled rosarin, rosavin, and rosin production in natural root culture and rosarin and rosavin production in hairy root culture. Approximately 80 and 95 % of the glycosides were released into the medium for natural and hairy root cultures, respectively. The highest intracellular rosavin yield 9.8 mg/g DW and total (intracellular + extracellular) 505 mg/l, was observed in hairy root culture when cinnamyl alcohol was applied on the day of inoculation with the addition of sucrose on the 14th day of culture. For rosin production, supplementation with CA alone on day 14 was more favourable with the highest intracellular amount 5 mg/g DW in natural root culture. Only traces of rosarin were detected.

## Conclusions

The discovery of multiple pharmacological activities of *Rhodiola* plants, in the majority *R. rosea*, and the increasing demand for *Rhodiola* preparations led to extensive research on the chemical profiling of the *Rhodiola* extracts and searching for a stable supply for plant material. The biological properties of *Rhodiola* extracts were initially attributed to the presence of salidroside which was first isolated from *Salix triandra* in 1926 (Brown et al. 2002). Salidroside could be produced synthetically on the multikilogram scale (Shi et al. 2011) or could be obtained in amounts which often exceeded its content in *Rhodiola* plants from other botanical families i.e. Salicaceae, Ericaeceae. But salidroside proved not to be the only active component of the extracts. Apart from salidroside: rosavin, rosin and rosarin were believed to be important for the pharmacological effects of *Rhodiola* extracts. Further detailed chemical examination of *Rhodiola* extracts revealed approximately 140 compounds from different chemical groups exerting a variety of pharmacological activities (Panossian et al. 2010). Moreover the superior efficacy of total *Rhodiola* extract to its single ingredients was demonstrated (Sokolov et al. 1985, 1990; Skopińska-Różewska et al. 2008b).

Nowadays nearly all raw plant material comes from natural resources, areas with short vegetative seasons and low temperatures. The intensive collection of plants for commercial purposes is connected with the excessive exploitation of natural populations which have become threatened with extinction (Brown et al. 2002; Galambosi 2006; Kolodziej and Sugier 2012; Peschel et al. 2013).

To meet the increasing need for *Rhodiola* plants *R. rosea* was introduced to the field cultivation in Russia, Poland, Germany, Sweden and Finland (Galambosi 2006; Kolodziej and Sugier 2012), *R. kirilowii* in Poland (Krajewska-Patan et al. 2009) and *R. sachalinens* in northern China, however in the latter case it was reported that cultivated plants were very sensitive to root rotting diseases (Meng et al. 1994; Xu et al. 1999). Moreover, plants cultivated in the fields require at least 5–7 years to be ready for harvest and to achieve a composition comparable to the plants gathered from natural populations (Galambosi 2006; Kołodziej and Sugier 2012).

On the basis of available data in plants of *R. crenulata*, *R. kirilowii*, *R. quadrifida*, *R. rosea* and *R.* *sachalinensis* higher salidroside content was determined in plants collected from natural sources than those derived from field cultivation. The highest salidroside amount was detected in wild plants of *R. kirilowii* (2.5 %). However, there is no available data on *R. crenulata* and *R quadrifida* field cultivation.

Plant biotechnology offers the opportunity to obtain plant material containing the desired compounds, often in higher amounts than the mother plant. Moreover plant collection to initiate in vitro cell and/or organ cultures could be performed with respect to the actual laws protecting endangered species. The specific organ or tissue connected with the biosynthesis could be maintained in in vitro cultures to achieve the highest production of compounds of interest. Among the various biotechnological strategies applied to enhance salidroside accumulation in *Rhodiola* cell, and organ cultures the precursor feeding approach seemed to be the most efficient. In *R. sachalinensis* cell suspension cultures the salidroside content was ten times higher than in plants collected from natural sources. While the addition of growth regulators alone to the in vitro cultures did not result in a significant increase in salidroside production.

The presence of rosin and its derivatives were affirmed to wild plants of *R. rosea*, *R. quadrifida* and *R. sachalinensis*. In cultivated plants their presence was confirmed only in *R. kirilowii* and *R. rosea*, with higher amounts detected in *R.* *rosea*. In the in vitro cultures a significant enhancement in the production of rosin and its derivatives was observed when the cultures were fed with the precursor cinnamyl alcohol. In *R. rosea* compact callus aggregate cultures the observed rosin and rosarin content was even higher than in field cultivated plants while the rosavin level was five times lower. The developed hairy root cultures of *R. kirilowii* supplemented with cinnamyl alcohol addition exhibited a multiplied potential for the production of rosin and its derivatives in comparison to field cultivated plants.

The results presented in the review point out that biotechnological methods could be an attractive approach to produce salidroside, rosin and its derivatives at higher or at least comparable levels to wild or cultivated plants.

## Abbreviations

BA: 6-Benzylaminopurine
CA: Cinnamyl alcohol
CAD: Cinnamyl alcohol dehydrogenase
CCA: Compact callus aggregates
CCR: Cinnamoyl-CoA reductase
CNS: Central Nervous System
DW: Dry weight
IAA: Indole-3-acetic acid
IBA: Indole-3-butyric acid
KT: Kinetin
MS: Murashige and Skoog (1962) medium
NAA: Naphthaleneacetic acid
PAL: Phenylalanine ammonia-lyase
Phe: l-Phenylalanine
SA: Salicylic acid
TDZ: Thidiazuron
Trp: l-Tryptophan
TGase: Tyrosol-glucosyltransferase
2,4-D: 2,4-Dichlorophenoxyacetic acid
4CL: Hydroxycinnamic acid CoA-ligase
4-HPAA: 4-Hydroxyphenylacetaldehyde
Tyr: l-Tyrosine
TyrDC: Tyrosine decarboxylase
UDP: UDP-glucose:tyrosol glucosyltransferase
UGT: Uridine diphosphate dependent glucosyltransferase
